# Spinal Cerebrospinal Fluid Leakage in Spontaneous Intracranial Hypotension: An Intrathecal Gadolinium Enhanced MR-Myelography Study

**DOI:** 10.5334/jbsr.1877

**Published:** 2020-01-30

**Authors:** Hakan Cebeci, Cem Bilgin, Selman Candan, Aylin Bican Demir, Bahattin Hakyemez

**Affiliations:** 1 Department of Radiology, Selcuk University, Faculty of Medicine, Konya, TR; 2 Department of Radiology, Bursa Uludag University, Faculty of Medicine, Bursa, TR; 3 Department of Neurology, Bursa Uludag University, Faculty of Medicine, Bursa, TR

**Keywords:** contrast leakage, magnetic resonance, intracranial hypotension, intrathecal, gadolinium

## Abstract

**Objectives:**

In the present study, the authors presented the intrathecal gadolinium enhanced MR-myelography findings of patients with spontaneous intracranial hypotension.

**Materials and Methods:**

Intrathecal gadolinium enhanced MR-myelography (Gd-MR-myelography) examinations between October 2012 and September 2018 in patients having clinical and radiological findings of spontaneous intracranial hypotension were evaluated retrospectively. Sites and types of contrast leakages in 20 patients who met inclusion criteria were reviewed. All patients had undergone T1-fat suppressed sagittal images of cervical, thoracic and lumbar region after the off label intrathecal injection of 1 ml gadolinium-based contrast agent.

**Results:**

Patients (18 female, 2 male) are aged between 23 and 62 years-old (mean age: 41.1). Cerebrospinal fluid (CSF) leakages were cervical in 6 patients, thoracic in 5 patients, lumbar in 5 patients. One patient had leakage in multiple levels on both cervical and thoracic region and another patient on both cervical-thoracic and lumbar regions. No patients had adverse effects related to intrathecal injection of gadolinium.

**Conclusions:**

Gd-MR-myelography is effective imaging modality to reveal spinal CSF leakages in patients with spontaneous intracranial hypotension.

## Introduction

Spontaneous intracranial hypotension (SIH) is a troublesome and often-misdiagnosed disease characterized by persistent severe headache. It particularly affects young and middle-aged people. Headache in SIH classically deteriorates during standing and relieves when lying down. Cerebrospinal fluid (CSF) leakage is the most common cause of this disease. Leakage leads to decrease in CSF volume, dilatation of intracranial spinal veins and subdural effusions. Rarely, life-threatening subdural effusions may occur. Thus, accurate clinical and radiological diagnosis, and appropriate treatment strategy is mandatory. Lumbar epidural blood patches are commonly applied in patients resistant to conservative therapy. Targeted epidural blood patch, fibrin glue injections and surgical dural repair are treatment options needing correct localization of CSF leakage [1,2,3,4,5,6].

Some SIH cases exhibit atypical clinical findings [3]. Final diagnostic criteria for SIH were defined by the International Classification of Headache Disorders, 3rd edition (ICHD-3). This criteria was based on CSF pressure measurements and detection of spinal CSF leakage by imaging modalities [7].

In patients with suspected SIH, cranial magnetic resonance imaging (MRI) is usually performed as first-line examination. Intravenous gadolinium-based contrast material should be used while performing brain MRI for the diagnosis of SIH, because, diffuse pachymeningeal enhancement is the most common radiological finding detected in brain MRI of SIH patients. Other possible pathologies that may be found are brain sagging, dilatation of venous sinuses and subdural effusion [5,8]. Spinal imaging also plays a crucial role, not only for diagnosis of SIH, but it also guides treatment strategies. Several imaging techniques were described in the literature for demonstration of CSF leakage. These modalities include computed tomography (CT) myelography, intrathecal gadolinium enhanced MR-myelography (Gd-MRM) and unenhanced heavily T2 weighted MRI. CT myelography offers more anatomic detail, and is particularly superior in showing CSF-venous fistulas. Many authors propose CT myelography as a test to be chosen firstly in detecting CSF leakages [9]. Gd-MRM is another imaging technique allowing direct visualization of the spinal CSF leaks that have been reported in small series and case reports [10]. The sensitivity of Gd-MRM in this subset of patients has not been well studied Gd-MRM but the test appears safe, with only small doses of contrast used for myelography [1,11].

A three-dimensional (3D) imaging sequence known as T1 high-resolution isotropic volume excitation MR (THRIVE) has been developed and is used commonly in the abdomen and in musculoskeletal areas [12]. There is no other report in the literature regarding THRIVE sequence in Gd-MRM examinations.

This study aimed to present Gd-MRM findings for localizing CSF leakage in patients having SIH.

## Patients and Methods

### Patients

We retrospectively reviewed Gd-MRM of patients having clinical and radiological diagnostic criteria for SIH who visited our institution from October 2011 to September 2018. History of spinal surgery, epidural and/or spinal anesthesia and spinal trauma were exclusion criteria. All consecutive patients were recruited and no patient was excluded due to technical reasons. Institutional review board approved this study with waived informed consent. A search of radiology information system retrieved 20 Gd-MRM examinations of patients with relevant clinical and radiological findings of SIH.

### Gd-MRM procedure and image analysis

Intrathecal administration of gadolinium-based contrast material was executed with fluoroscopy guidance in lateral decubitus position. Written informed consent was obtained before intrathecal gadolinium injection. 1 ml non-diluted gadoterate meglumine (dotarem®) was injected intrathecally at L3-L4 in all patients under sterile conditions using a 22 G needle. No adverse effect related to intrathecal gadolinium injection was noted. Patients transferred to MRI scanner. Images were acquired at thirty minutes after injection and delayed images were obtained in patients for whom CSF leakage were not demonstrated.

Gd-MRM image acquisitions were performed at clinical 3 Tesla MR imaging system (PhilipsAchieva3T, Best, Netherlands). Our routine Gd-MRM protocol includes T1 weighted two dimensional spectral presaturation by inversion-recovery (SPIR) sequence images of the cervical, thoracic, and lumbar spine in the sagittal (TE, 8 ms; TR, 540 ms; 252 × 200 matrix; 5 echo train length; slice thickness, 3 mm; flip angle, 90°; cervical FOV, 20 cm; thoracic and lumbar FOV, 28 cm), axial (TE, 8 ms; TR, 926 ms; 252 × 200 matrix; 5 echo train length; slice thickness, 3 mm; flip angle, 90; cervical FOV, 20 cm; thoracic and lumbar FOV, 28 cm). The sagittal 3D-THRIVE images were additionally obtained using the following parameters: TE, 2.1 ms; TR, 4.9 ms; fip angle, 10°; matrix, 280 × 280; field of view, 20 cm; slice thickness, 1.25 mm; echo train length, 5. The isovolumetric multi-planar images were generated along axial and coronal planes using a slice thickness of 2-mm immediately at the operating console. Delayed imaging after 2 hours with the same protocol is performed if a definite leak is not seen on initial imaging. Images were transferred to a dedicated workstation (GE Advantage Workstation; GE Healthcare, Milwaukee, WI). Two neuroradiologists with 17 years and five years of experience analyzed Gd-MRM images with consensus for detecting leakage localization. First, images of SPIR sequence was evaluated blind to patients data, and one week later, THRIVE sequence was evaluated. Findings between the two sequences were compared.

The Statistical Package for the Social Sciences for Windows (Version 16.0; SPSS, Chicago, Ill) was used for statistical analyses.

## Results

Twenty patients were identified with Gd-MRM procedures performed during the study period from October 2012 through September 2018. All patients had clinical and brain MRI findings consistent with SIH. The mean age was 41 years (range, 23–62 years) and 90% of patients were female.

In all of the patients, the gadoterate meglumine entered the subarachnoid space at the spinal level and enhanced the subarachnoid space totally. Gd-MRM revealed objective CSF leakages in 18 of 20 patients. All CSF leaks were detected in both SPIR and THRIVE sequences. There was no statistical difference between two sequences for demonstration of CSF leaks. Demographic data of included patients and the results of diagnostic imaging and treatment data are summarized in Table 1. In seven patients, CSF fistula was limited to one level, eleven patients revealed CSF leakages on more than one level. No patient showed diffuse contrast leakage to paravertebral areas.

**Table 1:** Demographic data of patients, CSF leak sites, epidural blood patch application status and suspected CSF leak etiology.

Patient no	Age (yr)	Gender	CSF leak localization	Epidural blood patch	Suspected CSF leak etiology

1	47	F	T6–T8	–	Spinal diverticula T6 through T8
2	27	F	C6–T9	+	Unclear, suspected spinal diverticula C6- trough T9. CSF-pleural fistula
3	42	F	C2	–	Unclear, suspected posterior dural tear
4	36	F	C2	+	Unclear, suspected posterior dural tear
5	38	F	L5–S1	–	Unclear, CSF leak from right neural foramina.
6	48	F	T1–T4	+	Unclear, CSF leak from right neural foramina.
7	34	F	T1–T5	+	Unclear, suspected posterior dural tear
8	52	F	C2	–	Unclear, suspected posterior dural tear
9	36	F	Cervicomedullary junction	–	Unclear, suspected posterior dural tear
10	55	F	C2	+	Unclear, suspected posterior dural tear
11	62	F	T10–T12	+	Spinal diverticula T10 through T12
12	26	F	No leak determined		
13	23	F	L2–L5	+	Foraminal CSF leak
14	42	F	T1–T2	+	Foraminal CSF leak
15	43	F	L5–S1	+	Foraminal CSF leak
16	34	M	C5–T1 and L5–S1	+	Foraminal and transspinöz leak
17	39	F	L1–L2	+	Foraminal CSF leak
18	60	M	L4–L5	+	Foraminal CSF leak
19	54	F	C2	+	Unclear, suspected posterior dural tear
20	25	F	No leak determined		

No complication associated with lumbar puncture and intrathecal administration of gadoterate meglumine was recorded. Gross behavioral changes, neurologic impairment and changes in vital signs were not seen in any of patients.

## Discussion

SIH is a very well known pathological entity with a classical diagnostic triad of postural headache, diffuse pachymeningeal gadolinium enhancement on MRI and low opening CSF pressure. According to many recent reports, in some variants of SIH, one of these entities may lack [3]. Otherwise, low CSF opening pressure was once thought to be the defining characteristic of the condition, it is now evident that CSF pressure is normal in the majority of patients [5]. Clinical features of SIH include headache increasing with standing, nausea, vomiting, blurred vision, interscapular or nuchal pain, tinnitus, dizziness, nystagmus, ear fullness, photophobia and phonophobia [3].

With the advances in imaging modalities, it is well understood that CSF leakage is the causative factor for SIH. Conservative treatment approaches are first line treatment strategies in SIH. Unresponsive patients need epidural blood patch or surgical repair of dural tear. Precise localization of CSF leakage is crucial in these patient groups, both in diagnosing SIH and in determining the best treatment strategy. Evidence of CSF leakage was accepted as one of the main criteria for diagnosis of SIH according to the International Classification of Headache Disorders [3,7,11]. CSF leakage localization was exactly determined in 16 (80%) of 20 patients. In our series, no leakage was demonstrated in two patients at initial and delayed imaging at the second hour. Maybe these patients needed more delayed imaging at four or five hours of injection. In a study conducted by Albayram et al., Gd-MRM in two of 19 patients with SIH did not reveal CSF leakage [11]. Another study by Chadzen et al. demonstrated 9/24 CSF leakages in SIH patients [2]. Our results were similar with Albayram et al. while we found more CSF leaks than Chazen et. al.

Intrathecal injection of gadolinium-based contrast materials is classically used for demonstration CSF leakages in rhinorrhea and otorrhea. Various reports revealed the safety of intrathecal gadopentetate dimeglumine injection at small doses [13,14,15,16]. However, gadoterate meglumine was the gadolinium-based contrast material chosen as intrathecal contrast agent in this study. Fewer reports are existing in the literature demonstrating safety of intrathecal gadoterate meglumine injection [17].

CT myelography is a reliable method for localizing level of CSF fistula. Mokri et al. reported 67% sensitivity for precise localization of leak, while sensitivity of spinal MRI was 50% and radionuclide scintigraphy was 55% in the same study [3]. However, more recent studies regarding efficiency of Gd-MRM for demonstrating CSF leaks reported higher sensitivity with Gd-MRM than CT myelography [1,2,10,11]. Also, CT myelography has a disadvantage of ionizing radiation exposure. Particularly, demonstration of slow flow CSF fistulas needs delayed image acquisition and increases radiation exposure. Gd-MRM technique has the advantages of avoiding ionizing radiation exposure and has a higher sensitivity to detect leaks compared to CT myelography. Thus, we do not use CT myelography in routine clinical practice, and we could not compare the sensitivity of CT myelography and Gd-MRM. We objectively detected leakage of CSF from subarachnoid space to epidural and/or paravertebral space in 18 patients. The results of this study showed 90% sensitivity of Gd-MRM for detecting CSF leakages in patients with SIH. This rate is similar with previous reports in the literature [1,10,11].

In seven patients, we detected CSF leakage in only one site or one level. In eleven patients, there were multi-level CSF fistulas. Single level leaks were reported 10/17 by Albayram et al. [11]. Our results showed higher rates of single level leaks. In patients with a single level leak, the localization of the leak was cervical in six cases and lumbar in 1 case. There was no thoracic leak site in single level dural tears. In patients with multiple level leaks, leakage was thoracic in five and lumbar in five patients. In one patient both lower cervical and upper thoracic area were the leakage sites. Our results revealed, single level leaks are mostly in cervical regions while multi-level leaks are mostly seen in thoracic and lumbar areas. In the literature, Albayram et al. could not localize the leak site in three of 19 patients because of diffuse contrast leakage to thoracic and lumbar area. High flow volume fistulas are thought to be the reason for the diffuse leaks and earlier image acquisition or intrathecal gadolinium injection in MRI unit may solve this problem. In our series, no diffuse contrast leakage was seen in patients included in this study.

**Figure 1:**
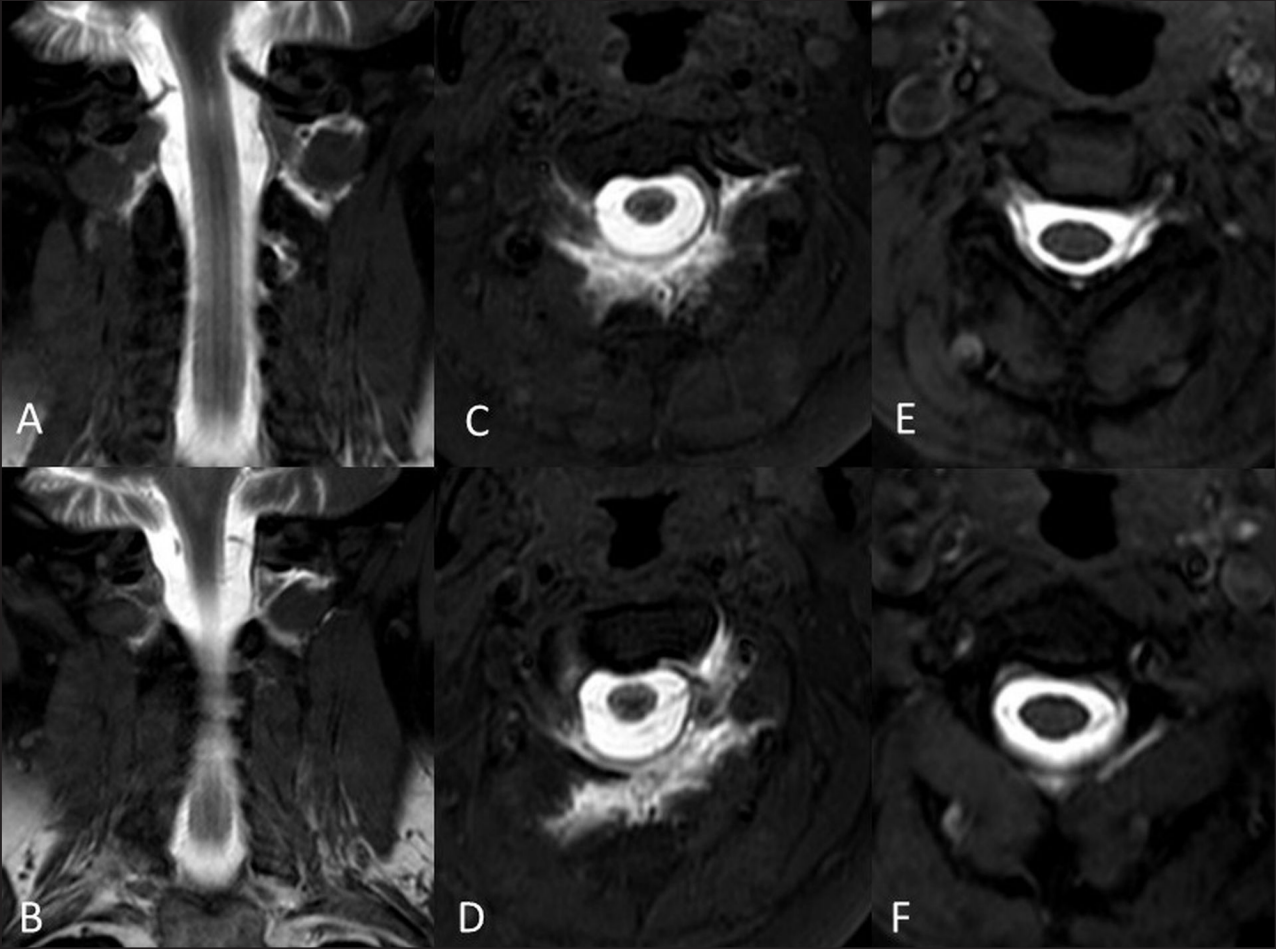
Intrathecal Gd-MRM images in a 52-year-old female patient demonstrated CSF leak at C2 level, T1W-SPIR coronal **(A, B)**, and axial **(C, D)**. CSF leak was decreased significantly on post-treatment T1W-SPIR axial images **(E, F)**.

Spinal meningeal diverticulas are well-known causes of CSF leakages. In a study by Chazen et al. comparing Gd-MRM and CT myelography, in six patients with meningeal diverticulas, CSF leakage was only detected with Gd-MRM. There were meningeal diverticulas in four of 20 patients in the present study and all meningeal diverticulas were in the thoracic region (Figure 1).

Degenerative disc diseases and osteophytes were another cause of CSF leakage in SIH patients [5]. We observed CSF leaks associated with degenerative changes in only two patients. Various previous studies in the literature reported disc hernia associated dural tears are more commonly in lower cervical and thoracic areas [18,19,20]. In our series, CSF leaks in two patients with disc hernia were in the cervical region.

In a 27-year old female patient in our series, left subarachnoid-pleural fistula inducing SIH and recurrent pleural effusion was detected. Small numbers of case reports are existing in the literature that reports CSF-pleural fistulas as a cause of SIH [21].

Intrathecal administration of gadolinium-based contrast agents is commonly applied to show contrast leakages in patients suffering from rhinorrhea and otorrhea. Various reports executed high diagnostic sensitivity of this technique in demonstrating CSF leaks in patients with SIH [1,14,22,23]. Gadopentate dimeglumin (Magnevist®) is the most common used gadolinium-based contrast agent in intrathecal injections. While various studies reported administration of gadodiamide (Omniscan®) and gadobutrol (Gadovist®) in small doses. We used 1 ml gadoterate meglumine to reveal spinal CSF leakage. To our knowledge, in the literature, there is only one report, conducted by Ozturk et. al., existing in literature that evaluated intrathecal injection of gadoterate meglumine [17,24].

Gd-MRM images of cervical, thoracic and lumbar area of patients included both SPIR and THRIVE sequences. THRIVE sequence is a T1-weighted gradient echo MRI sequence that allows for dual half-scan, improves fat suppression and enables fast dynamic scanning with submillimeter in-plane resolution, resulting in high contrast resolution [12]. To our knowledge, there is no other study existing in the literature that yields using THRIVE sequence in Gd-MRM. This study performed a blind comparison between two MRI sequences. The results did not exhibit a significant difference for demonstration of CSF leaks in SIH patients. All CSF leaks were detected by both SPIR and THRIVE sequences. Further investigations are needed with more cases to evaluate the efficiency of THRIVE sequence and comparison of sequences.

**Figure 2:**
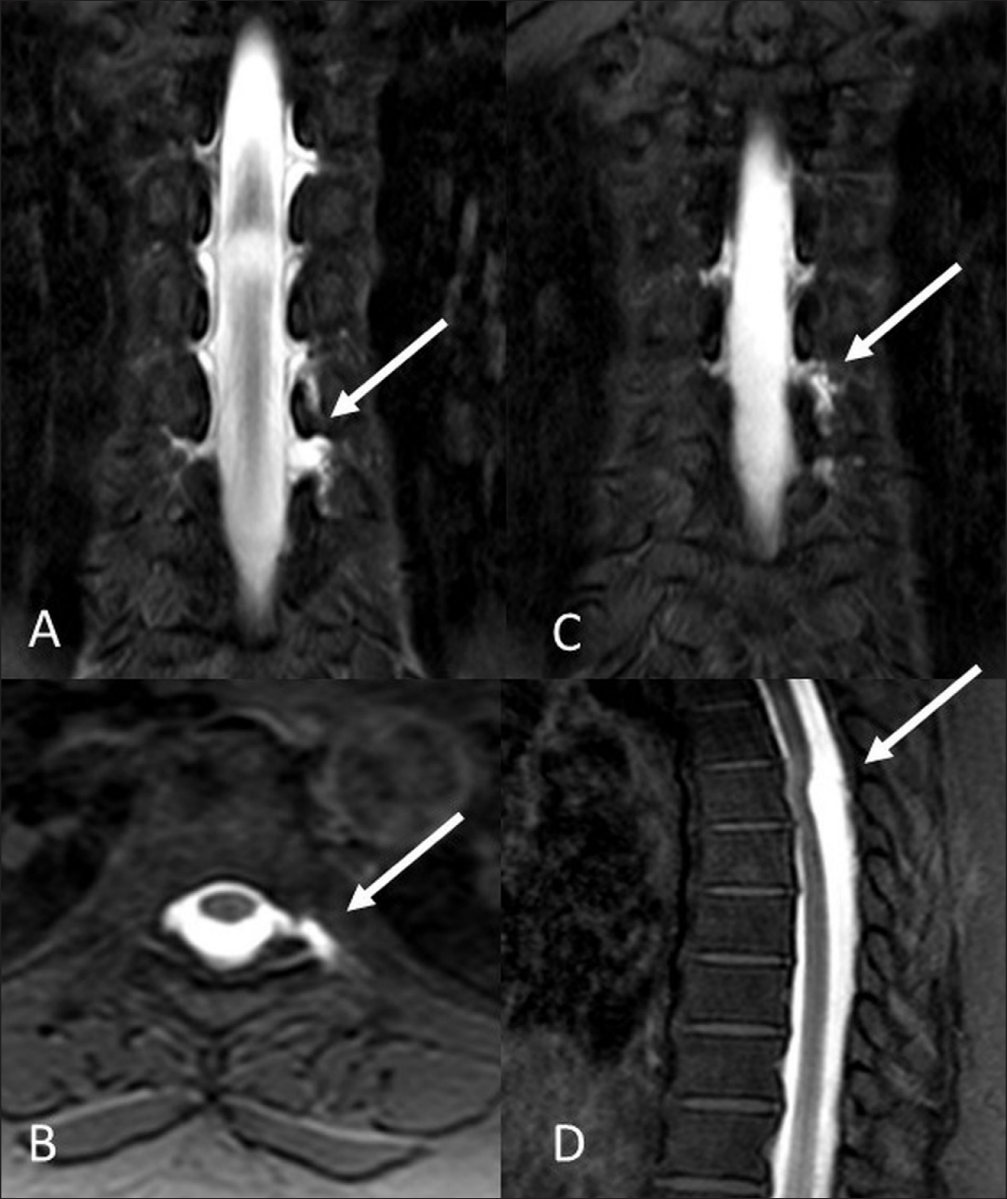
Intrathecal Gd-MRM images in a 47-year-old female patient demonstrated CSF leak at T6–T8 levels. T1W-SPIR coronal **(A, B)**, and axial **(C)** images revealed diverticular pouching and CSF leakage. A cystic lesion posterior to spinal cord was revealed in T1W-SPIR sagittal images **(D)**.

Treatment in SIH patients includes conservative therapy, epidural blood patch and surgical repair of dura in resistant CSF leaks. In our patients, 13 of 18 CSF leaks were treated with epidural blood patch. In a 52 year-old female patient with CSF leak at C2 level, multiple site epidural blood patch was applied through a single lumbar entry with a microcatheter. Thirty ml autologous blood introduced to cervical epidural areas. Control Gd-MRM images revealed significant decrease in CSF leakage and complaints of the patient resolved (Figure 2).

Our study has several limitations. The retrospective study design and relatively small number of patients is the first limitation. Second, intrathecal administration of gadolinium-based agents in this study group constitutes off-label use and is not currently approved worldwide. Long term follow-up effects of injected intrathecal gadolinium are not well known yet.

## Conclusions

In conclusion, Gd-MRM is an effective and safe, minimally invasive technique for detecting CSF leakages in patients with SIH. It provides multiplanar imaging capabilities without risk of radiation exposure. It is more sensitive than other imaging modalities lsuch as CT myelography and radionuclide cisternography. However, studies regarding long-term patient follow-up for intrathecal gadolinium-based contrast material are needed to detect delayed complications.
